# An Even Fairer Exchange: Further Reasons Why Living Kidney Donors in England Should Be Financially Compensated

**DOI:** 10.1007/s11673-024-10420-5

**Published:** 2025-06-03

**Authors:** Richard C. Armitage

**Affiliations:** Academic Unit of Population and Lifespan Sciences, School of Medicine, University of Nottingham, Clinical Sciences Building, Nottingham City Hospital Campus, Hucknall Road, Nottingham, NG5 1PB UK

**Keywords:** Kidney transplantation, Organ donation, Compensation, Medical ethics

## Abstract

Rodger and Venter have proposed a monopsony system in which the National Health Service (NHS) in England, as the single buyer, allows living kidney donors to opt-in to receive £35,000 tax-free financial compensation while preserving the right to donate without such compensation. This approach aims to alleviate the severe and growing shortage of kidneys available for transplant in England and is projected to generate substantial economic savings for the NHS. This paper sets out to strengthen their proposal by: (1) presenting updated figures on the increasing kidney transplant wait list in England to highlight the urgency for intervention; (2) detailing the rigor of the existing donor evaluation process to mitigate concerns about exploitation and coercion in compensated living donation; (3) outlining the various kinds of living kidney donation and the U.K. Living Kidney Sharing Scheme, to demonstrate that the proposal’s projected economic benefits are likely to be underestimations; (4) suggesting five modifications to the proposal that do not significantly alter its underlying structure; and (5) providing additional arguments against the major objections to such proposals—that financial compensation is exploitative, coercive, and likely to “crowd out” altruistic donors—and showing how the five suggested modifications could strengthen the proposal by bolstering it against those objections. The paper strengthens existing arguments for a pilot project of financial compensation for living kidney donors in England.

## Introduction

Rodger and Venter (2023) propose a monopsony system—in which the National Health Service (NHS), as the single “buyer,” continues to distribute kidneys according to clinical priority—to address the shortage of kidneys available for transplant in England. The authors build on the suggestion of Erin and Harris (2003) by proposing that kidney donors in England could opt-in to receive financial compensation (a tax-free £35,000 being the suggested figure), whilst still preserving the right of individuals to donate without receiving any compensation. The approach, should it be adopted, is argued to save hundreds of lives each year, lessen the burden on dialysis services, significantly reduce the kidney transplant wait list time, save financial resources that could be redirected to preventing kidney disease, provide fair financial compensation for costs to the donor (such as the morbidity and mortality risks associated with nephrectomy, postoperative pain and discomfort, uncertainty about the long-term health impacts of nephrectomy, and concerns about whether the donated kidney might be needed by a relative or friend in the future), and help to raise awareness of living donation. Finally, the authors address three objections that are commonly raised against proposals of financially compensated living donation—that such compensation is exploitative, coercive, and likely to “crowd out” altruistic donors—and argue that careful implementation of financially compensated living kidney donation would mitigate these concerns. Based on these arguments, the authors recommend piloting financial compensation for living kidney donors at a transplant centre in England.

I am supportive of the proposal of Rodger and Venter and am largely in agreement with their reasoning. In this paper, I aim to strengthen their proposal in a variety of ways: first, I shall provide updated figures pertaining to the kidney transplant wait list in the United Kingdom to highlight the urgency with which an intervention to increase the number of available kidneys is required; second, I shall detail the rigour of the existing donor evaluation process, an understanding of which should mitigate some concerns over the risks of exploitation and coercion in compensated donation; third, I shall outline the various kinds of living kidney donation and the U.K. Living Kidney Sharing Scheme and use this to reveal how Rodger and Venter’s projections of the economic benefits of compensated donation are likely to be underestimations; fourth, I shall suggest five modifications to the proposal, which serve to strengthen it without significantly altering its underling structure; fifth, I shall provide further reasons, in addition to those offered by Rodger and Venter, for why the three major objections raised against their proposal are insufficient and explain how the five suggested modifications could strengthen the proposal by further bolstering it against those objections.

Admittedly, it is highly likely that Rodger and Venter have thought deeply about many, if not all, of the points I will raise in this paper and were only prevented from discussing them by the requirement for brevity in academic paper. Nevertheless, what follows should be regarded as an attempt to further demonstrate the strengths of the authors’ proposed system, and to potentially improve upon it.

## Kidney Transplant Waitlist in the United Kingdom

Rodger and Venter outlined the current bleak state of the kidney transplant waitlist in the United Kingdom. Unfortunately, the situation has continued to worsen since their paper was published.

The number of patients registered on the kidney or kidney and pancreas transplant wait list in the United Kingdom increased from 3,525 in March 2021 to 5,023 in March 2022, to 5,655 in March 2023, and to 6,250 in March 2024 (the number is now substantially higher than that prior to the COVID-19 pandemic) (NHSBT 2024b, 2023, 2022b). In each of these years, between 275 and 304 patients died while awaiting kidney transplantation and between 460 and 625 were removed from the waitlist because their condition deteriorated to such a degree that transplantation became unfeasible.

As highlighted by Rodgers and Venter, the kidney waitlist continues to increase despite improvements to transplant infrastructure, education, and the adoption of opt-out systems of organ donation. Because only around 1 per cent of those who die each year in the United Kingdom are eligible to donate their organs (NHSBT 2024), it is becoming increasingly necessary to consider alternative approaches to increase the number of available kidneys for transplantation. The need to reopen the debate about financially compensating living kidney donors in England is, therefore, becoming increasingly urgent.

## Donor Evaluation in Living Kidney Donation in the United Kingdom

Rodgers and Venter briefly mention the practices and safeguards that are currently standard practice in living kidney donation in the United Kingdom. It is worth detailing these features to communicate the rigour of the existing donor evaluation process which, should the proposal be adopted, would be simply expanded to include living kidney donors who are financially compensated. Understanding the thoroughness of this process should mitigate some concerns over the risk of exploitation and coercion in compensated donation.

The primary goal of the living kidney donor evaluation process is to ensure the suitability of the donor and to minimize the risks of donation. The process involves identifying contraindications to donation and potential clinical risks (both physical and psychosocial) by subjecting the donor to an evidence-based protocol with multi-disciplinary input and discussion (BTS 2018). Investigations are undertaken in a particular sequence that protects the donor from unnecessary (especially invasive) procedures only if and until the donor progresses to the relevant stage of the process. The evaluation consists of a rigorous medical assessment (including urine and blood investigations, electrocardiogram, chest X-ray, and radioisotope measurement of renal function, in addition to any supplementary investigations that are required to assess the suitability of an individual donor), surgical assessment (including CT or MR imaging of the kidneys and their vasculature), detailed counselling from the renal medical and transplant surgical teams, and clinical psychology or psychiatric assessment (BTS 2018). All donors must also undergo an interview with an Independent Assessor who is trained and accredited by the Human Tissue Authority (HTA) and acts on behalf of the HTA to ensure that the donor has the capacity to consent, has in fact provided valid consent in the absence of duress or coercion, and that reward is not a factor in the donation (this final point would, of course, need to be altered to allow donors to receive the proposed compensation, while all other forms of compensation outside the proposed regulated monopsony would continue to be unlawful and would be assessed for by the Independent Assessor) (BTS 2018; HTA 2018; Human Tissue Act 2004). Only if the Independent Assessor is satisfied on each of these criteria will approval for living donation be provided.

The process of donor evaluation generally takes place over a protracted series of months, which safeguards against donors making impulsive or ill-conceived decisions to donate which they might eventually regret. The donor is able to withdraw from the donation process at any time, and it is the primary responsibility of the donor assessment team to support them in this decision if they choose it (BTS 2018).

## Kinds of Living Kidney Donation and the U.K. Living Kidney Sharing Scheme

Spain has remained the world leader in the rate of deceased organ donation for many years (Streit et al. 2023; Rada 2024). In 2023, there were 49.38 deceased donations per million people (pmp) in Spain, 22.35 deceased donations pmp in the United Kingdom, 9.20 living donors pmp in Spain, and 14.18 living donors pmp in the United Kingdom (IRODaT 2023). On December 31, 2023, there were 4,790 patients on the Spanish organ transplant waiting list (La Moncloa 2024), and 6,250 patients on the U.K.’s kidney or kidney and pancreas transplant list alone in March 2024 (NHSBT 2024). If the U.K. deceased donation rate was increased to that of Spain (rounded up to 50 pmp) without reducing the rate of living donation, 3,385 deceased organ donations would take place each year (U.K. population = 67.7 million [IRODaT 2023]). When combined with the 938 living donors per year (NHSBT 2024), the resulting 4,323 transplants would lie substantially short of the 6,250 patients in the United Kingdom who are awaiting a transplanted kidney or kidney and pancreas alone. Accordingly, even if deceased donation rates in the United Kingdom could be increased to those of Spain without negatively impacting the rates of living donation, thousands of patients would still require transplantation, suggesting that additional measures to increase the rate of living donation are required. In addition, the outcomes of living donation are superior to those of deceased donation, meaning living donation is considered the gold standard (Nemati, et al. 2014).

Rodgers and Venter’s paper deals with living kidney donation in England, but explores neither the existing infrastructure of living donation, nor the various kinds of living donation that take place, in the country. These are worth outlining because some of their features could be harnessed to further bolster the arguments made in Rodgers and Venter’s paper.

Four kinds of living kidney donation take place in the United Kingdom: directed donation, paired-pooled donation, directed altruistic donation, and non-directed altruistic donation.

Directed kidney donation is the kind of donation that immediately leaps to mind when the idea of living organ donation is invoked. It involves a living individual donating a kidney to a specified individual with whom they have a prior relationship, such as a relative or friend.

Living kidney donations can be “shared: across the United Kingdom through the U.K. Living Kidney Sharing Scheme (UKLKSS) (NHSBT 2017). This contains linked donor–recipient pairs which each consist of an individual in need of a kidney (recipient) and another individual (a loved one) who is willing to donate (donor), but the pair’s tissue incompatibility prohibits a donation between them. Through the UKLKSS, incompatible linked donor–recipient pairs are “matched” (through quarterly “matching runs”) with other such pairs that, in some combination, are collectively compatible for kidney sharing. In paired-pooled donations, two-way (paired donation) sharing occurs between two linked pairs, while three-way (pooled donation) sharing occurs between three linked pairs. Around 250–300 recipients partake in each quarterly matching run, around 25 per cent of which are new patients partaking for the first time. Since 2012, about 15 per cent of recipients have received a donated kidney after partaking in one matching run, about 34 per cent after four, about 57 per cent after eight, and about 64 per cent after 10 (NHSBT 2022).

Living donation is termed “altruistic” if two conditions are satisfied: first, the donor has no prior relationship with the recipient; second, the donor has not agreed to donate an organ for a loved one to receive an organ from another donor in return (such as in paired-pooled donation). Altruistic donation is either directed or non-directed (NHSBT 2021): in directed altruistic donation, the kidney is donated to a specified individual with whom the donor has no prior relationship (such as the subject of a media campaign); in non-directed altruistic donation, the kidney is donated to an unspecified individual with whom the donor has no prior relationship. Both kinds of donation are considered “altruistic” because the donor has no prior relationship with the recipient and has not formed a linked donor–recipient pair with a loved one. Within the UKLKSS, non-directed altruistic donors can donate to recipients in linked donor–recipient pairs to trigger altruistic donor chains consisting of up to three donations per chain (NHSBT 2017). The first non-directed altruistic kidney donation in the United Kingdom took place in 2006, and 357 took place between the beginning of the UKLKSS in 2012 and January 2024 (NHSBT 2024c).

## Economic Benefits

Rodgers and Venter note that, in 2009–2010, £1.45 billion was spent on treating chronic kidney disease in England. Updated statistics pertaining to the United Kingdom reveal that the total direct costs of diagnosed chronic kidney disease and kidney replacement therapy (such as dialysis) in 2022 amounted to 4.48 billion US$ (£3.35 billion) (Chadban et al. 2024). Assuming these costs were proportional to the population size of the home nations, around £2.83 billion was spent in England (84.5 per cent of the 2022 U.K. population lived in England)(ONS 2024). As such, these costs have almost doubled in just over a decade and are projected to undergo further substantial increase by 2027 (Chadban et al. 2024).

Incorporating their suggested tax-free £35,000 financial compensation for donation, the authors project a total cost saving of ~ £186,000 per kidney transplant patient over a ten-year period. As the compensation would be available to all living donors, this projection would apply to each of the four kinds of living kidney donation. However, this might be an underestimation in the kinds of donation that involve organ sharing as made possible by the UKLKSS.

Many recipients are able to identify willing donors and form linked donor–recipient pairs to allow kidney sharing in paired-pooled donations and altruistic donor chains. Furthermore, many linked donor–recipient pairs must enter multiple consecutive matching runs before finding compatible matches to allow organ sharing (NHSBT 2022). This suggests that the limiting factor to kidney sharing is not the availability of willing donors per se but the availability of willing *compatible* donors. It is possible, therefore, and perhaps even likely, that few of the donors in linked donor–recipient pairs would opt-in to receive financial compensation if it became available. It is simultaneously likely that, if the availability of financial compensation does increase the pool of available kidneys, it mostly increases the non-directed altruistic kind of donation, since many recipients are currently able to form linked-donor pairs in the absence of available compensation. If this is the case, altruistic donor chains of up to three donations might take place with only the initiating non-directed altruistic donor choosing to opt-in to receive financial compensation. Crucially, the donations in altruistic donor chains are unlikely to take place (at least without a significant delay) without being “unlocked” by a non-directed altruistic donor, the number of which is likely to increase with the availability of compensation.

Accordingly, using the authors’ estimated ~ £186,000 total cost saving per kidney transplant patient over a ten-year period (which includes £35,000 donor compensation), the total cost saving for a three-transplant altruistic donor chain over a ten-year period in which only the non-directed altruistic donor chooses to opt-in would be ~ £628,000, or an average ~ £209,000 per kidney transplant patient in the chain over a ten-year period. These savings are substantial, greater than those initially predicted, and likely to be compounded further by the increased number of altruistic donor chains made possible by the greater prevalence of non-directed altruistic donors if compensation is made available. As called for throughout the authors’ paper, a pilot project of financial compensation for living kidney donors is required to test these predictions.

## Suggested Modifications to the Proposal

While I am supportive of Rodger and Venter’s proposal, it could be strengthened by a combination of the following five modifications without significantly altering the proposal’s underling structure: first, a mandatory long-wait period from the point of “applying” to donate a kidney to the point of donating it, such as one or two years; second, a mandatory long-wait period from the point of donating a kidney to the point that financial compensation is collected, such as one or two years; third, provide financial compensation as in-kind benefits rather than a cash lump sum, such as through alterations to the donor’s tax code or contributions to the donor’s pension account; fourth, apply an “income floor” for compensated donation that excludes all those, for example, whose annual earnings are in the U.K.’s lowest quintile (which would, by definition, exclude the poorest 20 per cent of donors); fifth, allow the donor to opt-in for the compensation to be directed to a charitable organization rather than to themselves. The potential utility of these suggested modifications shall be demonstrated in the remainder of the paper.

## Objections to Financially Compensating Kidney Donors

Rodgers and Venter address three commonly raised objections to proposals for financially compensated kidney donation, specifically in defence of their proposed regulated monopsony. In what follows, I shall provide further reasons for why these three major objections are insufficient when raised against the proposal and explain how the five suggested modifications could strengthen the proposal by further bolstering it against those objections.

### Financial Compensation is Exploitative

Claims have been made that a system which provides kidney donors with a financial payment would be exploitative of the poorest in society (Greasley 2014; Adair and Wigmore 2011; Danovitch and Delmonico 2008; Hughes 2009). Such individuals include those whose income places them below the poverty line, those who have suddenly lost a large amount of money, and those who face an approaching deadline for the repayment of a substantial debt, and who therefore reluctantly turn to financially compensated kidney donation in a state of financial desperation.

The majority of such concerns over exploitation emerge from the likely consequences of open, unfairly priced, unregulated markets of living kidneys. Such a market, of course, is far from the tightly regulated, fairly priced, country-bound monopsony proposed by Rodgers and Venter. However, even within a regulated monopsony, some worry that only the poorest will be motivated to donate and will do so only to ameliorate their dire financial situation. For example, Kate Greasley argues that those who donate in regulated markets “will self select on the basis of poverty and desperation” (Greasley 2014), meaning the proposal of Rodgers and Venter would still be exploitative. Greasley is also concerned that, because only the poorest will donate, and the NHS will distribute kidneys according to clinical priority, the wealthy will choose not to donate because kidneys will flow up a socioeconomic gradient from the poor to the wealthy, thereby widening socioeconomic health inequalities. These claims, however, ignore the hundreds of living kidney donations that take place in the NHS each year (907 in 2023–2024) without financial compensation (NHSBT 2024b) and which, therefore, are not undertaken by the donor as a means to escape poverty (in this regard, donors have been recognized as the only party in the donation process to not be paid or receive an important benefit in kind, which has itself been identified as exploitative) (Erin and Harris 2003; de Castro 2003). In addition, a study of 57,896 living kidney donors in the United States between 1998 and 2010 found that rates of donation increased in a stepwise manner across median household income quintiles (rates of donation were lowest in the lowest quintile and highest in the highest quintile) (Gill et al. 2013). Accordingly, while the poorest donors might be more likely to opt-in to receive financial compensation, they would not be the only group to donate per se. It is hard to make the case that the poorest donors accepting compensation and the wealthiest donors refusing the same compensation constitutes exploitation of the poor.

It has been noted that many people work in risky jobs in exchange for financial renumeration, yet we do not regard these jobs as impermissible on grounds of exploitation (Danovitch and Delmonico 2008; Greasley 2014; Gill and Sade 2002; Savulescu 2003). Risky jobs include combat roles in the armed forces, electric pylon engineers, fire fighters, lifeguards, police officers, and construction workers. For example, there were a total of 540 U.K. armed forces deaths due to hostile action in the Afghanistan and Iraq theatres between 2001 and 2021, and 2003 and 2011, respectively (MOD 2023), while 138 workers were killed in work-related accidents in 2023–2024 (fifty-one of which were in the construction industry and fifty of which involved falls from a height) (HSE 2024). Accordingly, it has been argued that, if we allow individuals to be financially compensated for choosing risky jobs that generate substantial social benefit (such as the nation’s defence and the creation of public infrastructure), we should similarly allow them to be financially compensated for the social benefit generated by their choosing to donate a kidney (Savulescu 2003) (while donating a kidney is not in itself a job, the social benefit that it generates, and the inherent risk of doing so, can be considered similar to social benefit and inherent risk of risky jobs). Greasley rejects this argument by claiming that, while no exploitative practice is justified, kidney donation is a sufficiently extreme case to render it impermissible (Greasley 2014). This line of reasoning again fails to recognize the hundreds of living kidney donations that take place in the NHS each year without financial compensation, which society permits and considers highly valuable. It is hard to argue that we should allow (or celebrate) uncompensated donation but prohibit the provision of compensation for exactly the same action.

Greasley also rejects the risky job analogy by evoking the asymmetry between the certain harms caused by the permanent loss of a kidney and the merely potential harms posed by risky jobs. In doing so, Greasley focuses exclusively on realized physical harms and ignores the certain psychological harms to both workers in risks jobs and their families (as is caused when, for example, a soldier is deployed to war). While a risky job is carried out over a longer duration (potentially over the course of a career), the inherent risk in such jobs occurs intermittently rather than being constantly present, such as when a solider is not actively deployed or when a pylon engineer is not scaling a pylon. There is no clear ethical reason why the social benefit afforded by the single risk-imbued act of compensated organ donation is meaningfully different to a prolonged risky job that, in most of its moments, poses little risk. It therefore seems that the permissibility of risky jobs that generate social benefit should be extended to compensated living kidney donation which similarly generates substantial society value.

The similar argument has been made that society allows individuals to engage in risky sporting activities (such as rock climbing, skiing, and downhill mountain biking) and that we should similarly allow individuals to donate a kidney and be financially compensated for doing so. Greasley finds this to be a poor analogy because these scenarios do not involve the sporting participant being exploited (Greasley 2014). However, in the United Kingdom, the negative health consequences of these pursuits (such as the fractures, head injuries, and visceral damage of major trauma) would be dealt with by the NHS, which is a publicly funded health system financed by general taxation. It could be argued, therefore, that the individual exploits the tax-payer by being harmed by participation in risky sporting activities. Accordingly, it seems unreasonable to regard these activities as permissible while simultaneously prohibiting compensated kidney donation on the grounds of exploitation.

It goes without saying that the rigorous process of donor evaluation that currently takes place in England will continue to be applied to all donors, including both those who opt-in to receive compensation and those who are poor. This should guard against concerns of exploitation of poor donors, since only donors with sufficient degrees of physical and mental health will continue to be permitted to donate, regardless of their financial status.

If concerns that Rodgers and Venter’s proposal is exploitative of the poor persist, then any or all of the first three suggested modifications could be introduced (mandatory long-wait periods prior to donating, mandatory long-wait periods after donating before the compensation is collected, and the provision of compensation as in-kind benefits rather than as cash lump sums). These modifications would prevent the process from constituting a means by which a significant sum of cash can be rapidly accessed and thereby protect those who have suddenly lost a large amount of money or face an approaching debt repayment deadline from potential exploitation.

Should concerns regarding exploitation of the poor, such as regarding those whose income places them below the poverty line, ultimately prove insurmountable—such as if compensated donation is considered an example of mutual advantageous exploitation rather than harmful (Wertheimer, 1987), then the fourth suggested modification could be introduced (applying an “income floor” for compensated donation that excludes the poorest donors). The suggested tax-free £35,000 is equivalent to approximately a £45,000 gross annual salary in the United Kingdom, which is substantially above the £33,000 median average U.K. salary. Accordingly, this figure is likely to be impactful for not only those in the lowest income quintile. Rodger and Venter proposed this figure as it is “slightly lower than the median lowest amount of financial compensation that was perceived as an undue inducement for family/friends and substantially lower than for a stranger in the United States” (Rodger and Venter 2023). Since rates of donation were found to be lowest in the lowest median household income quintile and highest in the highest median household income quintile in the United States (no equivalent U.K. data are currently available), excluding the poorest donors this is unlikely to substantially reduce total donations (note that the poorest donors would continue to be able to donate without receiving compensation). A challenging side-effect of this modification, however, would be the so-called “double bind,” as termed by Margaret Radin (1996), which argues that a state should not prohibit a means to escape poverty if it is unwilling or unable to change the conditions that lead to this poverty (albeit Radin did not specifically direct the term at compensated living organ donation). Concerns over the reasoning of the “double bind,” such as those of Greasley, are only relevant in open, unfairly priced, unregulated markets (such as analogizing a system that financially compensates kidney donation with wealthy multi-national corporations that knowingly underpay their employees who have no alternative job options available to them) or through doubt that a fair price for a donated organ would be achieved (Greasley 2014). These concerns do not apply to the tightly regulated and fairly priced monopsony proposed by Rodgers and Venter such that an income floor for compensated donation would risk the injustice of the resulting double-bind.

An additional apprehension regarding exploitation in financially compensated kidney donation concerns black markets. Concerns have rightly been raised over the harmful consequences of kidney black markets in the absence of a legally regulated system (Koplin 2018; Greasley 2014; Goyal et al. 2002). It has also been claimed, however, that even in regulated monopsonies that financially compensate kidney donation, wealthy individuals in need of an organ would offer supplementary payments to further incentivize donors (Semrau and Matas 2022). Such under-the-table payments are known to take place in Iran, which has operated a state-level market in kidneys since 1988 and remains the only country in the world to do so (Major 2008; Gordon and Gill 2013). The concern when applied to Rodgers and Venter’s proposal is that the ability for wealthy recipients to offer supplementary payments, in addition to the £35,000 compensation provided by the NHS, would undermine the monopsony in which the NHS is the single buyer and, in doing so, establish a black market in which the wealthy exploit the poor (Semrau and Matas 2022). Such concerns are invalid for at least three reasons: first, the legislative changes that would be required to permit the proposed regulated monopsony of compensated kidney donations would outlaw supplementary payments that take place outside this system, while the Independent Assessors who interview each donor are trained to identify such unlawful payments; second, if the proposed system does increase the number of available kidneys, the incentive and motivation for those in need of kidneys to provide supplementary payments would reduce (while the chance of the recipient receiving a kidney does increase if the specific donor would otherwise not donate in the absence of a supplementary payment, the magnitude of this increase is small, and vanishingly so if the proposed system substantially increases the number of available kidneys); third, the monopsony’s single buyer would continue to distribute kidneys according to clinical priority, meaning a recipient who provides a specific donor with a supplementary payment is no more likely to receive that specific donor’s kidney than the kidney of any other donor.

#### Financial Compensation is Coercive

Claims have been made that a system which provides kidney donors with a financial payment would be coercive (Caplan and Rhodes 2022; Koplin 2018; Rippon 2014). Rodgers and Venter argued that their proposal to allow donors to opt-in to financial compensation would not lead individuals being coerced into donating because the current practice in which all donors undergo an interview with an Independent Assessor from the HTA who is trained to recognize coercion would continue once financial compensation becomes available. They also argue that, if their proposed monopsony system does increase the number of available kidneys as intended, this would reduce the incentive and motivation for those in need of kidneys, or those acting on their behalf, to coerce individuals into donating, since the chance of identifying a compatible match has already been elevated by the proposal. This is in addition to the rigorous process of donor evaluation that would continue to be applied to all donors and would exclude donors with insufficient physical and mental health—including those who are being coerced to donate while in, or even because of their, poor states of physical or mental health—from donating.

In addition to these safeguards against potential coercion of donors, any or all of the first three suggested modifications could be introduced (mandatory long-wait periods prior to donating, mandatory long-wait periods after donating before the compensation is collected, and the provision of compensation as in-kind benefits rather than as cash lump sums) to protect the donor against potential coercion. While coercive force could be unceasingly applied throughout the duration of the long-wait periods (which would amount to years), the protracted length of time both reduces the chance of this occurring and increases the chance of it being detected, while the absence of a cash lump sum payment would entirely eradicate the coercer’s incentive.

Simon Rippon argues that, if financial compensation for living kidney donation became available, those living in poverty may be subjected to legal pressure to donate to generate the cash that is required to make their necessary payments (such as rent) or social pressure to donate to receive compensation rather than relying on public welfare payments (Rippon 2014). Going further than this, Julian Koplin argues that the poor stand to lose from the introduction of financially compensated living donation regardless of whether they choose to participate in it or not, because such widening of choice sets also changes the context in which decisions are made (Koplin 2018). Koplin worries that the availability of financially compensated living donation would cause indirect harm to those who choose not to donate, such as by moneylenders considering kidneys as collateral and offering less favourable terms on loans, and the psychological harms of the criticisms of relatives for deciding not to donate and thereby allowing the family to remain in poverty. However, the first three suggested modifications to the proposal would also prevent these indirect harms on those who choose not to participate: in-kind compensation would not provide the donor with a cash lump sum with which rent and loan repayments could be made, while the mandatory long-wait periods would prevent compensation being regarded as an alternative to public welfare payments (which are needed by the poor on an immediate and regular basis) and would protect against the criticisms of relatives (who presumably hope for an immediate lump sum with which to escape their poverty). If these concerns regarding coercion of the poor persist, they would be eradicated by the fourth suggested modification of applying an income floor for compensated donation that excludes the poorest donors whether they wish to participate or not.

### Financial Compensation Will “Crowd Out”

It has been claimed that financially compensating living donation would “crowd out” altruistic donors and reduce the overall number of available organs (Koplin 2018; Rothman and Rothman 2006)(note that, here, “altruistic” refer to uncompensated donation rather than the kinds of donation available within the UKLKSS). Rodgers and Venter argue that their proposal is unlikely to generate this outcome, however, as the total sum of compensation they propose is sufficiently large to overcome the crowding out effect. They also highlight that, rather than their proposal providing compensation by default, the donor must opt-in to receive it, thereby preserving the opportunity to be an “altruistic” donor and further reducing the risk of crowding out.

The fifth suggested modification (to allow the donor to opt-in for the compensation to be directed to a charitable organization rather than to themselves) is likely to further guard against the crowding out of “altruistic” donors. As it stands, the proposal provides donors with two options—either opt-in to receive compensation for donation or do not opt-in and go without compensation. The fifth suggested modification would provide the donor with a third option. The selected charitable organization could work on, for example, the prevention of chronic kidney disease, improving aftercare for transplant recipients, or expanding access to kidney replacement therapy and transplantation in parts of the world that currently lack it (although the chosen charity could work on a problem that is unrelated to kidney disease and the donor would be free to direct their compensation to the charity of their choice). Rather than crowding out “altruistic” donors, this third option is likely to crowd such donors *in*, as it would allow them to compound the altruism of their donation beyond the donation itself, which is likely to be an appealing feature for donors who are motivated by altruistic principles (the mandatory long-wait period need not apply if a charitable organization, rather than the donor, will receive the compensation, as the donor will not receive it at any point in such situations).

## Conclusion

This paper is supportive of the proposal of Rodger and Venter for a regulated monopsony system of compensated kidney donation to address the shortage of kidneys available for transplant in England and aims to further strengthen the proposal. As the number of individuals waiting to receive a kidney donation increases, so does the urgency with which a novel intervention to increase the number of available kidneys is required. The intervention proposed by Rodger and Venter, especially when combined with the existing UKLSS, is likely to generate substantial economic benefits for the NHS. The three major concerns raised against proposals for systems such as Rodger and Venter’s—that financial compensation is exploitative, coercive, and likely to crowd out—are shown to be insufficient by highlighting existing evidence, the expansion of the rigorous donor evaluation process to compensated donors, and a combination of five suggested modifications to the proposal. Accordingly, this paper strengthens existing arguments for a pilot project of financial compensation for living kidney donors in England, to test the predictions that such an intervention will alleviate the severe and growing shortage of kidneys available for transplant in the country and generate substantial economic savings for the NHS.
